# Children with Heart Conditions and Their Special Health Care Needs — United States, 2016

**DOI:** 10.15585/mmwr.mm6738a1

**Published:** 2018-09-28

**Authors:** Meng-Yu Chen, Tiffany Riehle-Colarusso, Lorraine F. Yeung, Camille Smith, EdS, Sherry L. Farr

**Affiliations:** ^1^Epidemic Intelligence Service, CDC; ^2^Division of Congenital and Developmental Disorders, National Center on Birth Defects and Developmental Disabilities, CDC.

Children with heart conditions often use more health care services and specialized care than children without a heart condition (*1*); however, little is known about the number of U.S. children with heart conditions and their special health care needs. CDC used data from the 2016 National Survey of Children’s Health (NSCH) to estimate the prevalence of heart conditions among U.S. children aged 0–17 years, which indicated that 1.3% had a current heart condition and 1.1% had a past heart condition (representing approximately 900,000 and 755,000 children, respectively). Sixty percent and 40% of children with current and past heart conditions, respectively, had one or more special health care needs, compared with 18.7% of children without a heart condition (adjusted prevalence ratios [aPRs] = 3.1 and 2.1, respectively). Functional limitations were 6.3 times more common in children with current heart conditions (30.7%) than in those without heart conditions (4.6%). Among children with current heart conditions, males, children with lower family income, and children living in other than a two-parent household had an increased prevalence of special health care needs. These findings highlight the importance of developmental surveillance and screening for children with heart conditions and might inform public health resource planning.

Heart conditions in children can be congenital or acquired and range from asymptomatic to life-threatening. Congenital heart defects (CHDs) are the most common type of birth defect in the United States, affecting approximately 1% of live births (*2*). Children with CHDs often use more health care or educational services than do children without CHDs and might require specialized care (*1*,*3*,*4*). Less is known about the prevalence or needs of children with acquired heart conditions. Previously, there have been no known U.S. population-based estimates of the number of children with heart conditions or their special health care needs.

NSCH is a population-based, nationally representative survey of parents or primary caregivers (parents) of noninstitutionalized U.S. children aged 0–17 years.* NSCH asks parents about a selected child’s health, health care access, and family characteristics. In 2016, a total of 364,150 households were sampled; 138,009 (37.9%) parents completed screener surveys, and 50,212 (36.4%) of those completed topical surveys. The overall weighted response rate was 40.7%.^†^

Parents were asked if they had ever been told by a health care provider that their child had a heart condition. Those who responded affirmatively were asked if their child currently had a heart condition. Children’s heart condition status was categorized as “current,” “past,” or “none.” Parents were also asked about their child’s special health care needs using a standardized five-item screener that included 1) need for or use of medications (other than vitamins) prescribed by a doctor; 2) need for or use of medical care, mental health, or educational services beyond those of a similarly aged child (referred to as “average use”); 3) limitation in the child’s ability to do things most children of the same age can do; 4) need for or use of specialized therapies such as physical, occupational, or speech therapy; and 5) need for or receipt of treatment or counseling for an emotional, behavioral, or developmental problem. If any special health care need was attributable to a medical, behavioral, or other health condition that had lasted, or was expected to last, 12 months or longer, the child was considered to have a special health care need. The questionnaire also inquired about 26 other health conditions.^§^

The numbers and percentages of children with current, past, and no heart conditions were calculated. Chi-square tests were used to examine the differences in demographic characteristics (sex, age, race/ethnicity, family income as a percentage of the federal poverty level [FPL], highest parental education level achieved, health insurance type, and household structure); other health conditions; and special health care needs, by heart condition status. Marginal prediction approach to logistic regression was used to assess the association between heart condition status and one or more special health care needs, adjusted for demographic characteristics. Among children with a current heart condition, characteristics associated with having one or more special health care needs also were examined. All analyses were repeated excluding children with Down syndrome or other genetic conditions because these children’s heart conditions might be related to the syndromes. All analyses included design parameters to account for complex sampling and weights to generate population-based estimates of the numbers and prevalences of children with and without heart conditions.

Among the 50,212 children in the sample, 1,733 (3.5%) were excluded from analysis because of missing information, including heart condition status (180), special health care needs (309), and demographic characteristics (1,244). Excluded children were more commonly nonwhite, not privately insured, and living in households with lower income, lower parental education level, and other than two parents than were children who were not excluded (p<0.05 for all). After weighting the data to represent the U.S. population of children 0–17 years, an estimated 900,000 U.S. children (1.3% of U.S. children; 95% confidence interval [CI] = 1.1–1.5) had a current heart condition, 755,000 children (1.1%; 95% CI = 0.9–1.3) had a past heart condition, and 68.1 million children (97.6%; 95% CI = 97.3–97.9) had no heart condition.

Among children with current heart conditions, 58.3% were male, 55.7% were non-Hispanic white, 21.5% had family income <100% of FPL, 64.8% had at least one parent with higher than a high school education, 72.3% lived in a two-parent household, and 55.4% had private health insurance (Table 1). Demographic characteristics did not differ by heart condition status. Among children with current and past heart conditions, 67.2% and 60.5%, respectively, had one or more other health conditions, compared with 46.7% of children with no heart condition (p<0.001).

**TABLE 1:** Characteristics of children aged 0–17 years, by parent-reported heart condition status — National Survey of Children’s Health, United States, 2016

Characteristic	Heart condition status						Chi- square p value
	Current		Past		None		
	Unweighted no.	Weighted % (95% CI)	Unweighted no.	Weighted % (95% CI)	Unweighted no.	Weighted % (95% CI)	
**Total**	**634**	**—**	**498**	**—**	**47,347**	**—**	**—**
**Sex**							
Male	356	58.3 (50.0–66.1)	267	53.5 (42.6–64.1)	24,189	50.8 (49.8–51.8)	0.17
Female	278	41.7 (33.9–50.0)	231	46.5 (35.9–57.4)	23,158	49.2 (48.2–50.2)	
**Age group (yrs)**							
0–5	185	28.9 (22.9–35.7)	136	27.9 (20.5–36.7)	13,717	32.4 (31.5–33.4)	0.16
6–11	194	44.0 (35.9–52.4)	144	32.7 (23.2–43.7)	14,139	33.9 (32.9–34.9)	
12–17	255	27.1 (21.6–33.5)	218	39.5 (29.1–50.9)	19,491	33.7 (32.8–34.6)	
**Race/Ethnicity**							
White, non-Hispanic	455	55.7 (47.3–63.8)	356	52.0 (41.0–62.8)	33,510	52.5 (51.5–53.6)	0.75
Other*	179	44.3 (36.2–52.7)	142	48.0 (37.2–59.0)	13,837	47.5 (46.4–48.5)	
**Family income as a percentage of federal poverty level^†^**							
<100%	72	21.5 (15.5–28.9)	58	28.7 (17.6–43.3)	4,309	20.5 (19.5–21.5)	0.28
100%–199%	112	27.4 (19.6–36.8)	81	19.4 (13.2–27.6)	7,375	21.9 (21.0–22.9)	
200%–399%	208	27.4 (21.8–33.9)	169	27.6 (20.6–36.0)	14,693	27.2 (26.3–28.0)	
≥400%	242	23.7 (18.7–29.7)	190	24.2 (17.6–32.3)	20,970	30.4 (29.6–31.2)	
**Parental education level** ^§^							
High school graduate or less	107	35.2 (26.9–44.6)	77	29.3 (19.9–40.9)	6,772	28.4 (27.3–29.6)	0.38
More than high school	527	64.8 (55.4–73.1)	421	70.7 (59.1–80.1)	40,575	71.6 (70.4–72.7)	
**Household structure**							
Two parents	503	72.3 (65.1–78.5)	393	77.6 (69.6–83.9)	38,606	75.8 (74.9–76.7)	0.54
Other	131	27.7 (21.5–34.9)	105	22.4 (16.1–30.4)	8,741	24.2 (23.3–25.1)	
**Insurance type** ^¶^							
Any private	459	55.4 (47.0–63.5)	354	50.7 (39.9–61.5)	36,679	61.6 (60.5–62.6)	0.10
Public, unspecified, or uninsured	173	44.6 (36.5–53.0)	141	49.3 (38.5–60.1)	10,544	38.4 (37.4–39.5)	

**Abbreviation:** CI = confidence interval.

* Includes Hispanic, non-Hispanic black, American Indian/Alaska Native, Native Hawaiian or Other Pacific Islander, and Asian.

^†^ Based on the U.S. Department of Health and Human Services Poverty Guidelines.

^§^ Highest education level among two parents or child's primary caregivers.

^¶^ 129 had missing information on insurance type.

Sixty percent of children with current heart conditions and 40.0% with past heart conditions had one or more special health care needs, compared with 18.7% of children without a heart condition (Table 2). Children with heart conditions most commonly needed or used prescription medicines (current = 42.8%; past = 26.6%) and had above average use of medical care, mental health, or educational services (current = 41.8%; past = 23.9%). Children with current or past heart conditions were 3.1 and 2.1 times more likely, respectively, to have one or more special health care needs than were children without a heart condition, with the largest relative differences observed for functional limitations (current aPR = 6.3; 95% CI = 5.0–8.1) (past aPR = 3.7; 95% CI = 2.4–5.6).

**TABLE 2:** Percentage and adjusted prevalence ratio* of special health care needs^†^ among children aged 0–17 years, by parent-reported heart condition status — National Survey of Children’s Health, United States, 2016

Special health care needs	Heart condition status				
	Current		Past		None
	% (95% CI)	aPR* (95% CI)	% (95% CI)	aPR* (95% CI)	% (95% CI)
Has one or more special health care needs	60.0 (51.6–67.8)	3.1 (2.7–3.6)	40.0 (29.9–50.9)	2.1 (1.6–2.7)	18.7 (18.0–19.5)
Needs or uses prescription medicines	42.8 (35.3–50.7)	3.0 (2.5–3.6)	26.6 (17.5–38.1)	1.9 (1.3–2.8)	13.8 (13.2–14.5)
Above average use of health care or educational services^§^	41.8 (34.5–49.4)	4.2 (3.5–5.1)	23.9 (17.2–32.2)	2.4 (1.8–3.3)	9.5 (9.0–10.1)
Has functional limitations	30.7 (24.3–38.0)	6.3 (5.0–8.1)	17.4 (11.5–25.5)	3.7 (2.4–5.6)	4.6 (4.1–5.0)
Needs or uses physical, occupational, or speech therapies	22.4 (16.9–29.0)	4.3 (3.2–5.7)	14.4 (9.2–21.8)	2.9 (1.8–4.6)	4.7 (4.3–5.2)
Needs or receives treatment or counseling for emotional, developmental or behavioral conditions	23.4 (17.8–30.0)	2.7 (2.1–3.5)	22.5 (15.9–30.9)	2.7 (1.9–3.8)	8.0 (7.5–8.5)

**Abbreviations:** aPR = adjusted prevalence ratio; CI = confidence interval.

* Prevalence ratio of special health care needs for current and past heart conditions versus no heart condition, adjusted for sex, age group, race/ethnicity, family income as a percentage of the federal poverty level, parental education level, and household structure.

**^†^** Based on having one or more of the following five conditions: needing or using prescription medicine; needing or using more medical care, mental health, or educational services than other children their age; having limitations in doing things, compared with other children their age; needing special therapy (e.g., physical, occupational, or speech therapy); or having an emotional, developmental, or behavioral problem in need of counseling or treatment. These conditions must be related to a medical, behavioral, or other health condition that has lasted or is expected to last 12 months or longer.

^§^ Beyond those of a similarly aged child.

Among children with current heart conditions, an increased prevalence of special health care needs was observed among males (aPR = 1.3; 95% CI = 1.1–1.7), children with family income <100% of FPL (aPR = 1.4; 95% CI = 1.0–2.0), and children living in other than a two-parent household (aPR = 1.3; 95% CI = 1.0–1.6) (Table 3). Findings did not change substantially after excluding 1,650 children with Down syndrome or other genetic conditions, 181 (11%) of whom had a heart condition.

**TABLE 3:** Associations between selected demographic characteristics and special health care needs among children aged 0–17 years who have a current heart condition — National Survey of Children’s Health, United States, 2016

Characteristic	One or more special health care needs	aPR* (95% CI)
	Weighted % (95% CI)	
**Sex**		
Male	68.9 (60.5–76.3)	1.3 (1.1–1.7)
Female	47.4 (34.5–60.7)	Referent
**Age group (yrs)**		
0–5	57.8 (45.9–68.9)	Referent
6–11	58.5 (42.7–72.7)	1.0 (0.7–1.2)
12–17	64.4 (53.4–74.4)	1.1 (0.9–1.3)
**Race/Ethnicity**		
White, non-Hispanic	62.4 (54.6–69.7)	Referent
Other^†^	56.8 (41.3–71.1)	0.9 (0.7–1.1)
**Family income as a percentage of federal poverty level** ^§^		
<100%	80.5 (67.3–89.3)	1.4 (1.0–2.0)
100%–199%	52.8 (32.6–72.2)	1.0 (0.7–1.5)
200%–399%	59.5 (47.8–70.2)	1.1 (0.9–1.5)
≥400%	50.1 (38.5–61.7)	Referent
**Parental education level** ^¶^		
High school graduate or less	62.0 (41.6–78.9)	1.0 (0.8–1.3)
More than high school	58.8 (51.6–65.7)	Referent
**Household structure**		
Two parents	54.2 (44.2–63.8)	Referent
Other	75.1 (63.3–84.0)	1.3 (1.0–1.6)

**Abbreviations:** aPR = adjusted prevalence ratio. CI = confidence interval.

* Prevalence ratios adjusted for sex, age group, race/ethnicity, family income, parental education level, and household structure.

^†^ Includes Hispanic, non-Hispanic black, American Indian/Alaska Native, Native Hawaiian or Other Pacific Islander, and Asian.

^§^ Based on the U.S. Department of Health and Human Services Poverty Guidelines.

^¶^ Highest education level among two parents or child's primary caregivers.

## Discussion

According to the 2016 NSCH, 1.3% and 1.1% of U.S. children had a current or past heart condition, respectively. Because the specific types of heart conditions were unknown (i.e., congenital versus acquired), comparing current findings with published estimates of CHDs or acquired heart conditions is difficult. The birth prevalence of CHDs is nearly 1%, and approximately 1 million U.S. children have CHDs (*2*). Although U.S. estimates of some acquired heart diseases such as those resulting from Kawasaki disease (*5*) and rheumatic heart disease (*6*) exist, the prevalence of other acquired heart conditions in children is unknown.

Children with CHDs are at increased risk for developmental disabilities and speech, motor, behavior, or learning problems (*1*), whereas the risk for children with acquired heart conditions has not been quantified. The higher prevalence of special health care needs among children with heart conditions, particularly functional limitations identified in this study, supports the American Academy of Pediatrics’ guidance on developmental surveillance and screening for early identification and intervention (*7*), particularly for children with complex CHDs (e.g. single ventricle defects) (*1*).

Similar to the present findings among children with CHDs, male sex, lower family income, and other than two-parent household structure have been associated with special health care needs in the general pediatric population (*8*). The differences in the prevalence of special health care needs by sex, family income, and household structure could reflect a difference in health status or differential ascertainment. Associations between special health care needs and family income and household structure might be attributable to stress and financial issues associated with the child’s health and treatment (*9*). More information is needed to know what resources might support families and benefit children.

The findings in this report are subject to at least five limitations. First, data are parent-reported and unconfirmed by medical records; however, according to findings from a study that used medical records to verify parental report of a diagnosis of autism (*10*), parental report of their child’s medical history might be valid. Second, separate analyses for congenital, acquired, or other heart conditions could not be conducted because information on the type of heart condition was not available. Third, the composition of heart conditions relies on what the responding parent considered a “heart condition” or a “current heart condition,” which might underestimate or overestimate the prevalence of heart conditions. Fourth, although the data were weighted for nonresponse, bias might remain. Finally, the temporality of special health care needs and family income or household structure is unknown.

These first population-based prevalence estimates of children with heart conditions and their special health care needs highlight the importance of guidelines for developmental surveillance and screening for early identification and intervention (*4*,*7*). These estimates could inform national and state child health programs to ensure that children with heart conditions receive necessary services.

**Summary**

**What is already known about this topic?**

Children with heart conditions often need specialized care. Little is known about the number of U.S. children living with heart conditions and their special health care needs.

**What is added by this report?**

In 2016, 1.3% of U.S. children had a current heart condition, and 1.1% had a past heart condition. Children with past and current heart conditions had higher prevalences of one or more special health care needs, compared with children without heart conditions.

**What are the implications for public health practice?**

These findings highlight the importance of developmental surveillance and screening among children with heart conditions for early identification and intervention and could inform public health resource planning.

## Footnotes

* https://mchb.hrsa.gov/data/national-surveys.

† https://www.census.gov/content/dam/Census/programs-surveys/nsch/tech-documentation/methodology/2016-NSCH-Methodology-Report.pdf.

§ Allergies, anxiety problems, arthritis, asthma, attention deficit disorder/attention deficit hyperactivity disorder, autism spectrum disorder, behavioral/conduct problems, blood disorders, brain injury, cerebral palsy, cystic fibrosis, depression, developmental delay, diabetes, Down syndrome, epilepsy/seizure disorder, headaches/migraines, hearing impairment, learning disability, mental retardation/intellectual disability, other genetic/inherited conditions, other mental health conditions, speech/language disorder, substance abuse, Tourette syndrome, and vision impairment.
